# Supplementary material for: Oligometastatic Prostate Cancer: A Comparison between Multimodality Treatment vs. Androgen Deprivation Therapy Alone
Source: Cancers (Basel). 2022 May 6;14(9):2313. doi: 10.3390/cancers14092313 (PMC9100905; doi:10.3390/cancers14092313)
Supplement: Supplementary file 1 [file cancers-14-02313-s001.zip › cancers-1510528-supplementary.pdf]

**Supplementary Table S1.** Adverse events related to treatment administration occurred in 74 oligometastatic prostate cancer patients stratified according to MMT vs. ADT alone.

| | ADT Alone | MMT |
|---|---|---|
| **Cardiac event** | 1 | 0 |
| **DVT/PE** | 1 | 0 |
| **Hematologic disorder** | 1 | 0 |
| **Pathologic bone fracture** | 4 | 4 |
| **Urinary acute retention** | 1 | 0 |
| **Ureteral stenting** | 2 | 0 |
| **Nephrostomy** | 1 | 0 |
| **TURP** | 4 | 0 |
| **Ileal resection** | 0 | 1 |

Abbreviations: androgen deprivation therapy (ADT), deep venous thrombus (DVT), multimodality treatment (MMT), pulmonary embolism (PE), trans-urethral prostate resection (TURP).
